# Corrosion Behavior of a Monolithic Zr-Cu-Al-Ag Bulk Metallic Glass and a Zr-Cu-Al-Ag Bulk Metallic Glass Matrix Composite in Sodium Chloride Medium

**DOI:** 10.3390/ma19173595

**Published:** 2026-08-24

**Authors:** Meng-Du Lyu, Huei-Sen Wang, Chih-Chun Hsieh, Mei-Hui Wu, Jason Shian-Ching Jang

**Affiliations:** 1 Department of Materials Science and Engineering, I-Shou University, Kaohsiung 84001, Taiwan; alger424@berlin.com.tw; 2 Product Application Technology Section, Steel & Iron Research & Development Department, China Steel Corporation, Kaohsiung 81233, Taiwan; 147884@mail.csc.com.tw; 3 Institute of Material Science and Engineering, National Central University, Taoyuan 32001, Taiwan; jscjang@ncu.edu.tw

**Keywords:** bulk metallic glass (BMG), pitting corrosion, passive film, precipitates, potentiodynamic polarization test

## Abstract

The corrosion mechanism and corrosion behavior of a monolithic Zr-based (Zr_48_Cu_36_Al_8_Ag_8_)Si_0.75_ bulk metallic glass (BMG) and a Zr-based (Zr_44_Cu_36_Al_8_Ag_8_Ta_4_)Si_0.75_ BMG matrix composite (BMGMC) in 3.5 wt.% NaCl solution were investigated. Potentiodynamic polarization tests were conducted to evaluate the corrosion and passive behavior of BMGs. Both monolithic BMG and BMGMC exhibited distinct pitting corrosion in sodium chloride solution. The monolithic BMG exhibited a higher value of pitting overpotential, η_pit_, and a wider passive region, when compared to that of BMGMC, indicating that the monolithic BMG has a better pitting resistance than the BMGMC. The worse corrosion resistance of BMGMC can be attributed to the weak passive film of the interface area between the precipitates and the glassy matrix, where it can be more easily broken through by halide ions, Cl^−^, preferentially. Furthermore, galvanic corrosion can occur due to the potential difference between Ta precipitates and the matrix of BMGMC, leading to an even more severe corrosion of the BMGMC.

## 1. Introduction

Multi-component bulk metallic glasses (BMGs) have attracted considerable attention for both scientific research and potential engineering applications because of their unique properties, which are often superior to those of conventional crystalline alloys [1,2,3]. Among them, Zr-based BMGs have been widely studied owing to their high glass-forming ability (GFA), wide supercooled liquid region, high strength, and high hardness. More recently, Ni-free Zr–Cu–Al–M BMG systems, where M represents alloying elements such as Ag, Ti, Pd, Nb, Y, or other minor elements, have received increasing interest [4,5,6,7,8,9,10]. These alloys are particularly attractive because they are composed of relatively environmentally friendly elements and exhibit high strength, improved ductility, and excellent GFA compared with many other Zr-based BMGs.

However, similar to most monolithic Zr-based BMGs, monolithic Zr–Cu–Al–M BMGs generally deform inhomogeneously below the glass-transition temperature [9]. This deformation behavior limits their plastic strain under compressive loading and restricts their broader structural applications. To overcome this limitation, bulk metallic glass matrix composites (BMGMCs) have been developed by introducing micro- or nano-scale ductile crystalline phases, such as Nb, Ta, or Hf, into the glassy matrix. These composites can be fabricated either by in-situ precipitation of crystalline phases within the BMG matrix or by ex-situ addition of reinforcing particles during melting, followed by casting into a glassy matrix [11,12,13,14,15]. Previous studies have shown that the precipitation of Ta phases in Zr-based BMG matrices can effectively improve ductility, leading to compressive plastic strains of approximately 15–30% [11].

For the practical use of Zr–Cu–Al–M BMGs and Zr–Cu–Al–M BMGMCs, their corrosion resistance in aggressive environments must also be carefully evaluated. In general, the presence of crystalline precipitates in a glassy matrix is often considered detrimental to corrosion resistance because such precipitates can introduce compositional or structural heterogeneities [12]. However, several studies have reported different behavior in Cu-based BMGMCs reinforced with Nb-, Mo-, or Ta-rich phases. After immersion in HCl, H_2_SO_4_, or NaCl solutions, these composite alloys exhibited better corrosion resistance than fully glassy monolithic Cu-based BMGs [12,14]. This suggests that the influence of crystalline reinforcements on corrosion resistance depends strongly on the alloy system, reinforcement phase, and corrosive environment.

The corrosion behavior of Zr-based BMGs has been examined in different corrosive environments, and previous studies have shown that these alloys may undergo localized attack when the passive film is damaged by chloride ions [12,15]. In recent years, the addition of alloying elements or second phases has also been used to modify the mechanical and corrosion properties of Zr-based BMGs [4,5,6,7,8,9,10]. Among these elements, Ta is attractive because of its high chemical stability, good corrosion resistance, and the formation of a stable Ta_2_O_5_ passive film. Owing to its limited solubility in Zr-based amorphous alloys, Ta can also form discrete Ta-rich particles within the glassy matrix and act as a reinforcing phase. However, the presence of Ta particles may introduce local chemical and electrochemical differences between the particles, their surrounding regions, and the amorphous matrix, which can influence the local corrosion response in chloride-containing environments.

Although the corrosion behavior of Zr-based BMGs has been widely studied, the role of Ta particles in the pitting corrosion of Zr–Cu–Al-based BMG composites remains unclear. The motivation of this study is to clarify how the introduction of Ta particles affects the localized corrosion behavior of Zr-based BMG composites, particularly the breakdown of the passive film and the development of pitting corrosion around Ta-containing regions. Accordingly, the Zr_48_Cu_36_Al_8_Ag_8_Si_0.75_ BMG and the Zr_44_Cu_36_Al_8_Ag_8_Ta_4_Si_0.75_ BMG composite were comparatively investigated in 3.5 wt.% NaCl solution using potentiodynamic polarization tests together with microstructural observations. The main objective was to determine the influence of Ta particles and their surrounding regions on passive-film breakdown, pit formation and propagation, and the subsequent localized corrosion behavior of the BMG composite.

## 2. Materials and Methods

The two alloy ingots used in this study were designed as a Zr-based (Zr_48_Cu_36_Al_8_Ag_8_)Si_0.75_ bulk metallic glass (BMG) and a Zr-based (Zr_44_Cu_36_Al_8_Ag_8_Ta_4_)Si_0.75_ BMG matrix composite (BMGMC). The BMGMC alloy was prepared from pure elemental Zr, Cu, Al, Ag, Ta, and Si with a purity of 99.9 wt.%. The constituent elements were weighed according to the nominal composition of Zr_44_Cu_36_Al_8_Ag_8_Ta_4_Si_0.75_ and arc-melted under a Ti-gettered argon atmosphere. Ta was introduced as an elemental alloying constituent rather than as pre-existing reinforcing particles or powder. After arc melting, the alloy was suction-cast into a water-cooled copper mold to obtain the BMGMC specimens. To form a homogeneous alloy ingot, the base alloy was produced through a two-step arc melting and casting process [11]. The bulk metallic glasses were alloyed by a vacuum arc melting furnace (VAM, TA-200, Materials Research Furnaces, LLC, Allenstown, New Hampshire, USA) at a cooling rate was below 10 K/s. After the melting was completed, the liquid alloy was suction cast into the water-cooled Cu mold to form a cast plate.

All BMG sample plates were then polished to remove existing oxides and the phase constitution of the as-cast plates were characterized using the X-ray diffractometer (XRD; Scintag X-400, Thermo Fisher Scientific, Santa Clara, CA, USA) with Cu Kα radiation with a wavelength of 1.5409 Å. The diffraction patterns were collected over a 2θ range of 20–80° at a scanning rate of 4° min^−1^. The surface observation and microstructure of BMGs samples using the scanning electron microscopy (SEM; Hitachi S-4700, Hitachinaka, Japan) operated at an accelerating voltage of 15 kV and a working distance of approximately 8–12 mm. The chemical composition was performed using an equipped with energy dispersive X-ray spectroscopy (EDS; HORIBA 7200-H, Tokyo, Japan). The initial microstructural characterization associated with the subsequent corrosion behavior, the Ta particle and its surrounding region were examined by transmission electron microscopy (TEM; Fei Tecnai G2, Hillsboro, OR, USA) before the corrosion test using foils extracted from specific sites using a 5 keV focused ion beam (FIB; SMI 3050, Seiko/SII NanoTechnology, Shizuoka, Japan). A low ion beam current of 40 pA was used for the final milling.

The corrosion behavior of the BMG alloys was investigated by electrochemical polarization measurements using a potentiostat/galvanostat (EG&G Princeton Applied Research, Model 273, Princeton, New Jersey, USA), with reference to the ASTM G5-14(2021) standard [16]. Prior to the electrochemical tests, the back of each specimen was connected to a conductive wire and embedded in epoxy resin, after which the exposed surface was ground with silicon carbide paper up to 2500 grit, degreased in acetone, rinsed with distilled water, and dried with compressed air. The polarization measurements were performed in a three-electrode cell, comprising the specimen itself as the working electrode, a platinum counter electrode and a standard saturated calomel reference electrode (SCE).

The electrolyte used was a 3.5 wt.% NaCl solution, which was deoxygenated by purging with N_2_ gas before each experiment was conducted and during the potential scan. This is to eliminate the interference of dissolved oxygen reduction reaction in the solution and to ensure that the measured current comes entirely from the electrochemical reaction of the metal itself (such as corrosion or passivation), thereby obtaining accurate data on the material corrosion characteristics. The potentiodynamic polarization curves of the specimens were measured, at a potential sweep rate of 1 mV/s, when the open-circuit potential (OCP) became almost steady after immersion in the electrolyte for at least 300 s. An excessively long immersion time could lead to severe pre-corrosion or pitting before the actual potential scan, which alters the initial surface topography. Therefore, a 300-s threshold was chosen as an optimized duration to achieve OCP stability while preventing significant pre-corrosion.

During the polarization scan, images of the specimen surface were observed and captured using the photographic device (Sony Digital Camera, ZV-E10, Tokyo, Japan). After electrochemical polarization testing, the corrosion morphologies and corrosion products formed on each specimen were characterized by SEM–EDS analysis.

## 3. Results and Discussion

### 3.1. Phase Constitution and Microstructure of the Monolithic BMG and BMGMC Alloys

**Figure 1:**
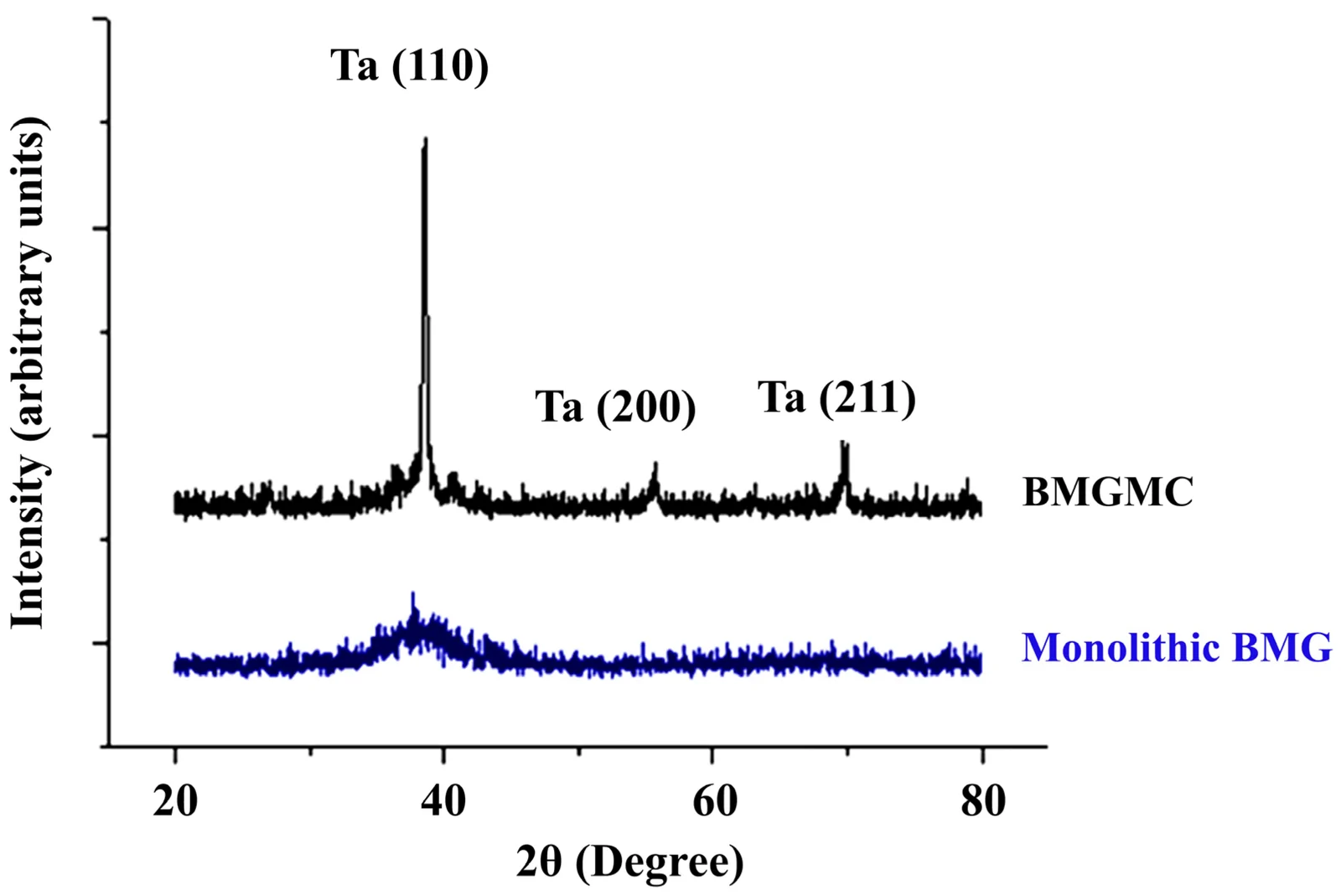
XRD patterns of the as-cast monolithic BMG plate and BMGMC.

Prior to the corrosion tests, the microstructures of the as-cast plates were examined. Figure 1 shows the XRD patterns of the monolithic (Zr_48_Cu_36_Al_8_Ag_8_)Si_0.75_ BMG and the (Zr_44_Cu_36_Al_8_Ag_8_Ta_4_)Si_0.75_ BMG matrix composite (BMGMC). For the monolithic BMG, a broad diffuse halo was observed in the 2θ range of 30–50°, indicating that the alloy matrix was predominantly amorphous. In contrast, the BMGMC sample exhibited not only a broad halo in the same diffraction range but also three sharp diffraction peaks corresponding to a body-centered cubic (BCC) crystal structure. These peaks indicate the precipitation of Ta particles within the amorphous matrix.

**Figure 2:**
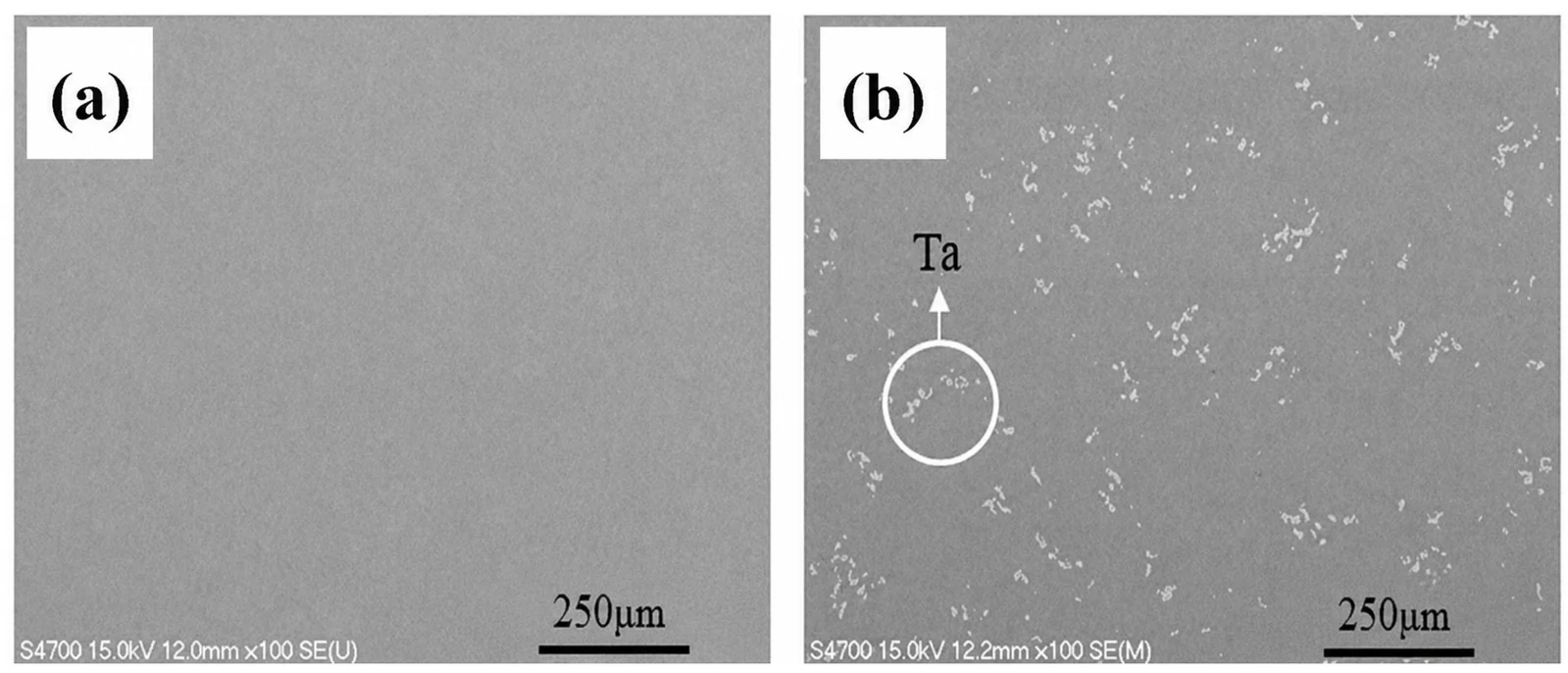
SEM images of the as-cast (**a**) monolithic BMG and (**b**) BMGMC, respectively.

SEM observations were further carried out to examine the microstructural features of the BMG and BMGMC samples, as shown in Figure 2a,b. No obvious contrast variation was observed in the matrix of the monolithic (Zr_48_Cu_36_Al_8_Ag_8_)Si_0.75_ BMG, as shown in Figure 2a, further supporting its amorphous nature. In contrast, after the addition of Ta, Ta particles with sizes of approximately 5–25 μm were observed in the amorphous matrix of the BMGMC sample, as shown in Figure 2b.

### 3.2. Potentiodynamic Polarization and Localized Corrosion in 3.5 wt.% NaCl Solution

**Figure 3:**
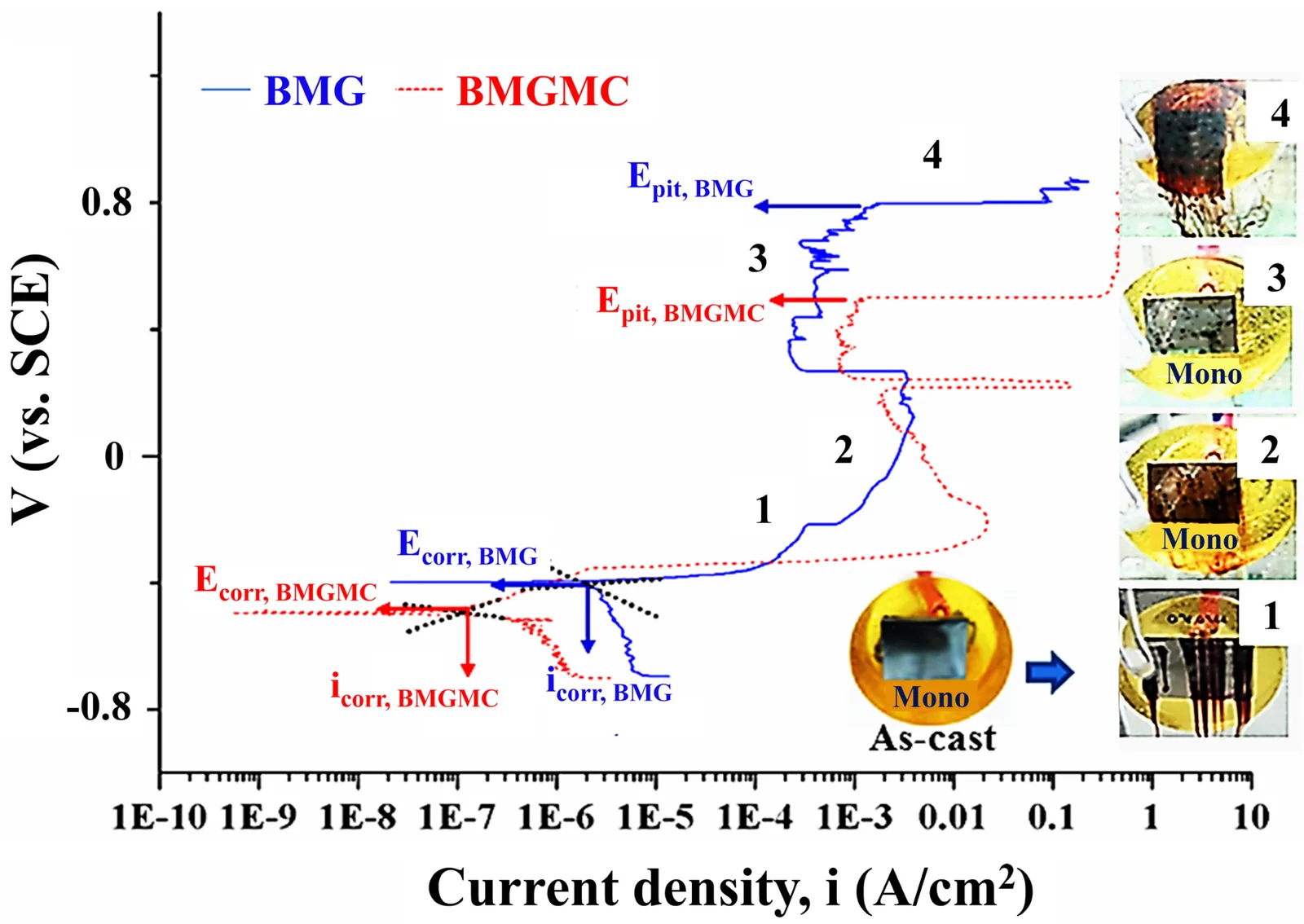
Polarization curves of the monolithic BMG and the BMGMC in 3.5% NaCl solutions at room temperature.

Figure 3 shows the potentiodynamic polarization curves of the monolithic BMG and the BMGMC in 3.5 wt.% NaCl solution at room temperature. Overall, the corrosion behavior of the BMGMC sample was similar to that of the monolithic BMG. Representative observations of the corrosion process of the monolithic BMG are also included in Figure 3. In the image marked “1”, the initiation of localized corrosion can be observed. As the electrode potential increased, the dissolution rate of the monolithic BMG increased rapidly, as shown in the image marked “2”, where a marked increase in both the number and size of corrosion pits was observed. With a further increase in potential, the monolithic BMG entered a passive state, as shown in the image marked “3”. At this stage, pitting corrosion was largely suppressed, and only a small amount of corrosion products was formed. However, when the applied potential exceeded the pitting potential (E_pit_), rapid and severe corrosion occurred, as shown in the image marked “4”.

As listed in Table 1, the E_corr_ value of the BMGMC alloy was −0.495 V (vs. SCE), which was more negative than that of the monolithic BMG alloy, −0.4015 V (vs. SCE). This result suggests that the BMGMC alloy has a higher tendency for spontaneous corrosion than the monolithic BMG alloy. For both monolithic Zr-based BMG and BMGMC alloys, a passive oxide film can form on the alloy surface due to the presence of valve metals such as Zr and Al [10,17,18,19,20,21]. However, when these alloys are exposed to aqueous solutions containing chloride ions, such as Cl^−^, corrosion may initiate at weak points in the passive oxide film, including scratches, defects, or other surface irregularities.

For the BMGMC alloy, previous study [22] has suggested that additional weak points may be associated with the interfaces between the precipitates and the glassy matrix. In these interfacial regions, the passive film is often distorted or less stable, making them preferential sites for chloride ion adsorption and attack. As a result, the BMGMC alloy is more likely to suffer localized corrosion in 3.5 wt.% NaCl solution.

However, the i_corr_ value of the BMGMC alloy was 1.2 × 10^−7^ A/cm^2^, which was approximately one order of magnitude lower than that of the monolithic BMG alloy, 2.8 × 10^−6^ A/cm^2^. This indicates that the BMGMC alloy exhibited a lower corrosion rate than the monolithic BMG alloy during the initial stage of polarization testing. This behavior suggests that Ta precipitates may play an important role in reducing the initial corrosion rate of the BMGMC alloy.

In NaCl solution, elemental dissolution generally proceeds through the formation of metal–chloride complexes [23,24]. The formation tendency of these complexes differs among alloying elements, resulting in different dissolution rates near the alloy surface. For the monolithic BMG and the BMGMC alloy used in this study, the main compositional difference lies in the Ta and Zr contents. The standard enthalpies of formation at 298.15 K for ZrCl_4_ and TaCl_5_ are −980.5 and −859.0 kJ/mol, respectively [25]. Although these values provide thermochemical information on the formation of the corresponding metal chlorides, they cannot be directly used to evaluate the corrosion tendency of Zr and Ta in an aqueous NaCl solution. Corrosion behavior is governed by electrochemical thermodynamics and kinetics, as well as by the stability and breakdown of the passive film. In this regard, the lower i_corr_ value of the BMGMC may be associated with the presence of Ta precipitates, since Ta exhibits strong passivation owing to the formation of a stable Ta-based oxide film. The Ta-rich regions may therefore contribute to reducing the overall corrosion current density near the corrosion potential. Nevertheless, this lower i_corr_ does not necessarily indicate improved resistance to localized corrosion, because breakdown of the passive film in the presence of chloride ions may still promote localized galvanic interactions between the Ta-rich precipitates and the surrounding Zr-rich glassy matrix.

As the electrode potential was further increased, both the BMG and BMGMC alloys entered a passive state. It should be noted that the passive region of the monolithic BMG was wider than that of the BMGMC alloy. In general, a wider passive region indicates a more stable and protective passive film [15]. Therefore, although the BMGMC alloy showed a lower initial corrosion current density, its narrower passive region suggests that its passive film was less stable under higher applied potentials, making it more susceptible to localized corrosion.

At higher applied potentials, both the monolithic BMG and BMGMC samples exhibited a sudden rise in anodic current density, which marks the onset of pitting corrosion and is defined as the pitting potential (E_pit_). The pitting overpotential (η_pit_ = E_pit_ − E_corr_) is therefore used to assess the resistance of the alloy to localized pitting corrosion [15].

### 3.3. Post-Corrosion Morphology and Chloride-Induced Localized Corrosion Mechanism

**Figure 4:**
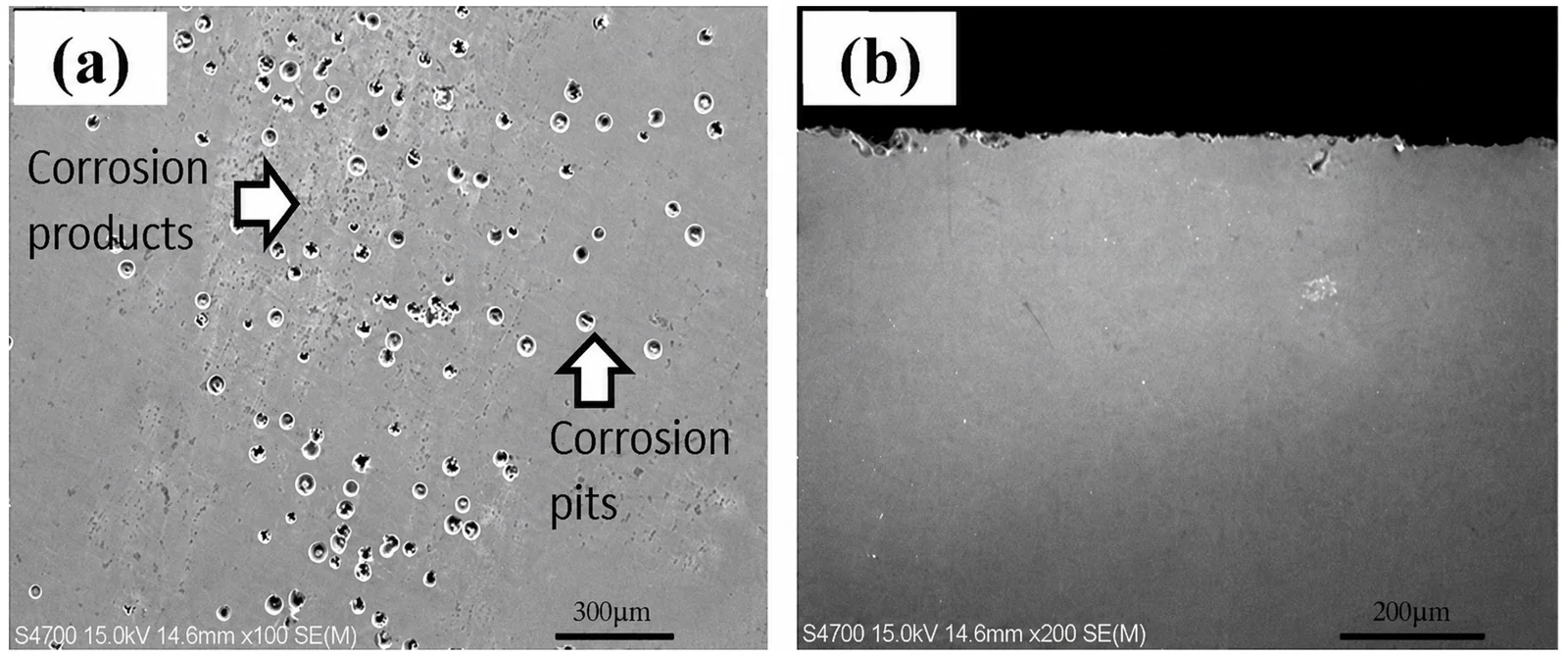
SEM images of the corroded monolithic BMG (**a**) at the surface and (**b**) the cross-section after the polarization tests.

**Figure 5:**
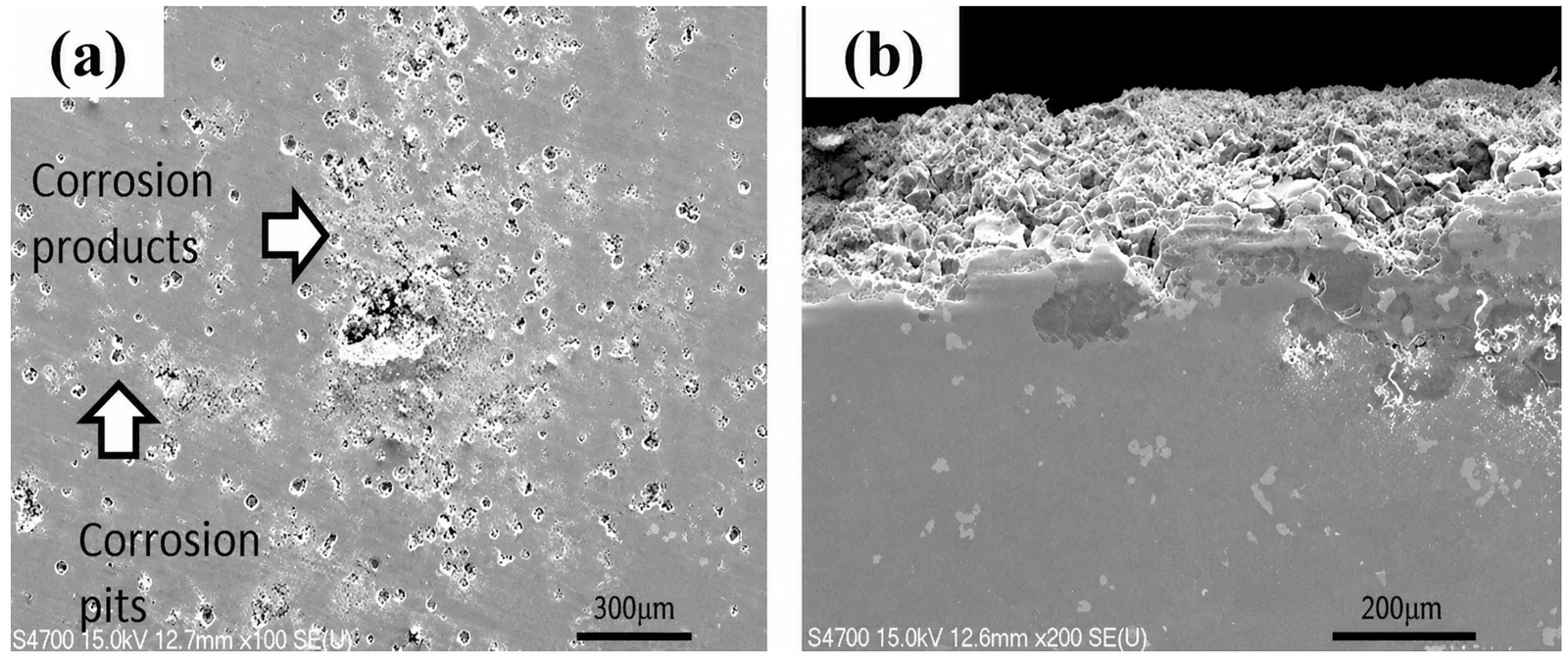
SEM images of the corroded BMGMC (**a**) at the surface and (**b**) the cross-section after the polarization tests.

A lower value of *η*_pit_ indicates a higher susceptibility to pitting corrosion, whereas a higher value suggests better resistance to localized attack. Compared with the BMGMC sample, the monolithic BMG exhibited a higher *η*_pit_ value. Both the wider passive region and the higher *η*_pit_ value indicate that the monolithic BMG possessed better resistance to localized pitting corrosion. Therefore, as shown in Figure 4 and Figure 5, the BMGMC sample suffered more severe corrosion damage and perforation after polarization testing than the monolithic BMG, which is consistent with the potentiodynamic polarization results discussed above.

**Figure 6:**
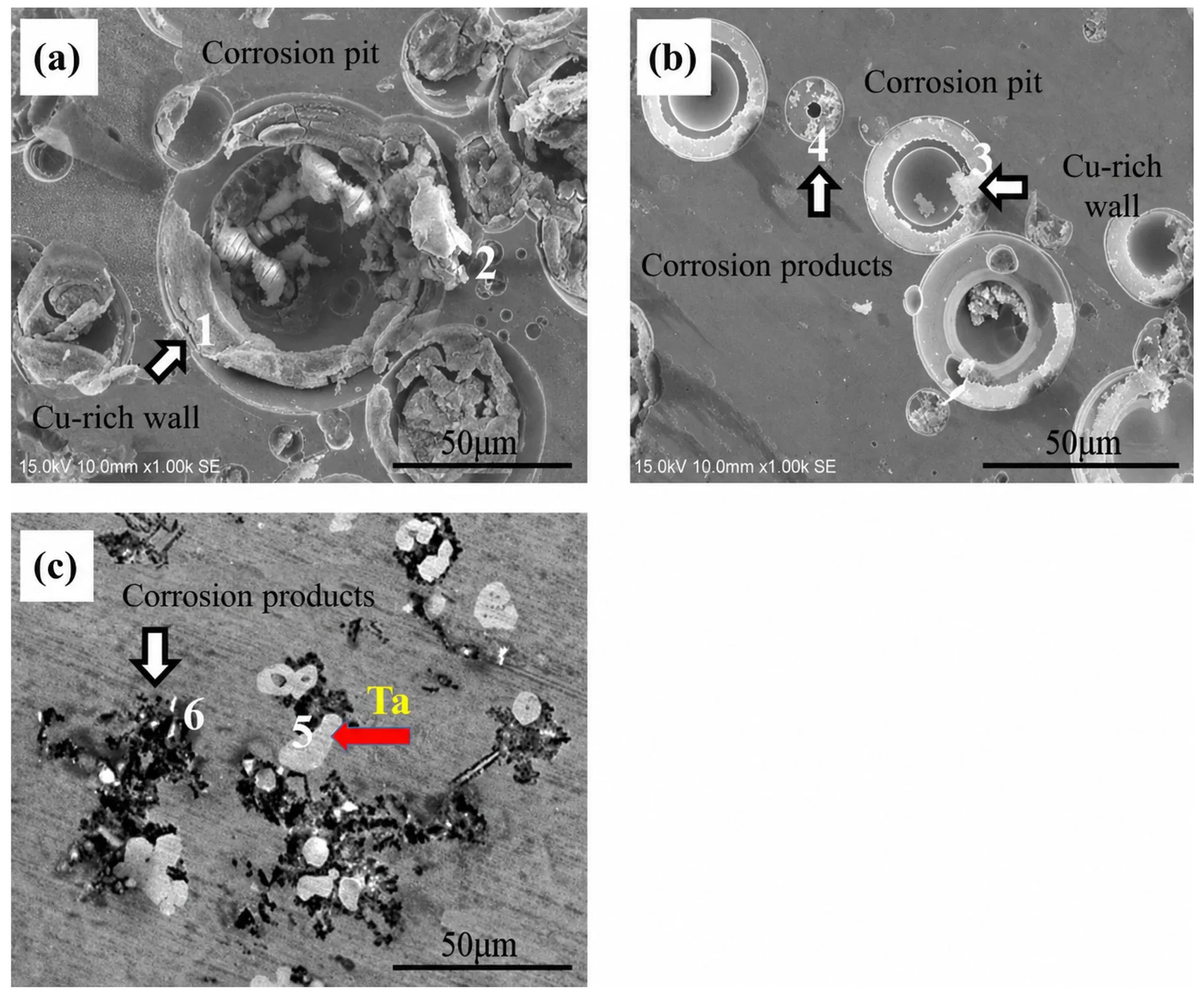
Higher magnification of the corrosion pits of (**a**) the monolithic BMG, (**b**) the BMGMC and (**c**) severe corrosion in the area adjacent to the Ta precipitates of the BMGMC.

**Figure 7:**
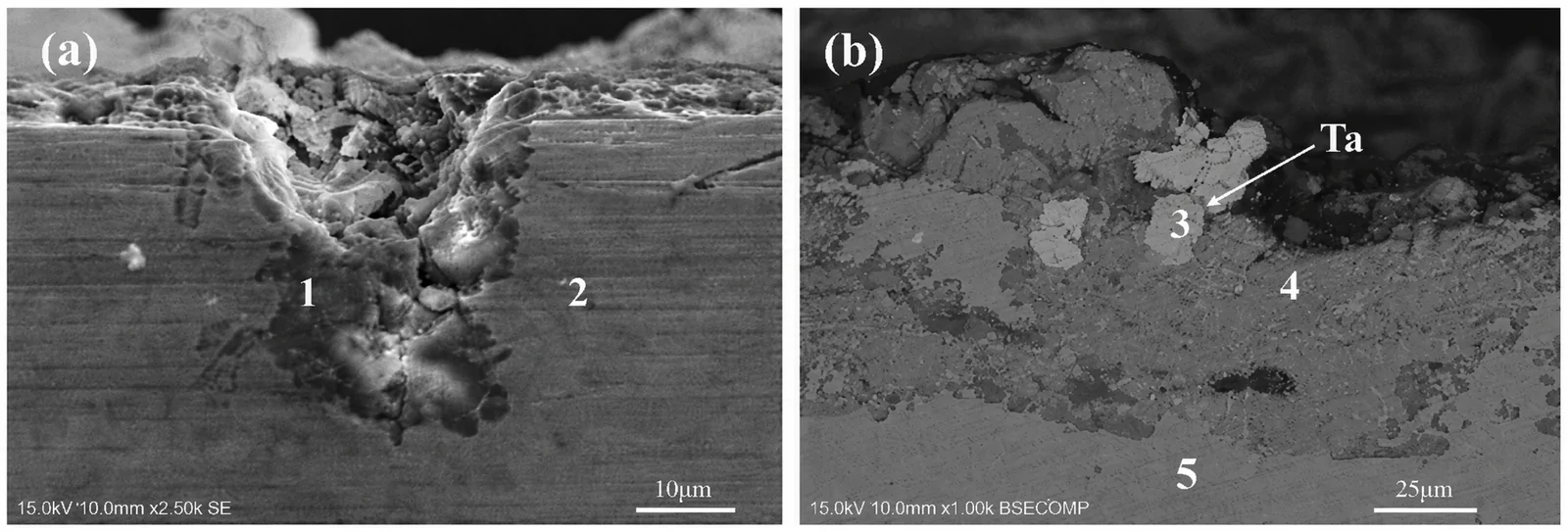
Higher magnification of the characteristic morphologies of the pits or corrosion products at the corroded cross-section of (**a**) the monolithic BMG and (**b**) the BMGMC.

To further clarify the chloride-induced localized corrosion mechanism of the monolithic BMG and BMGMC alloys, the surface and cross-sectional morphologies, as well as the corresponding corrosion products after polarization testing, were examined by SEM and EDS. Figure 6 and Figure 7 show the SEM images of the corroded surface and cross-section, respectively, while the corresponding EDS results are summarized in Table 2 and Table 3. Clear pitting corrosion was observed in both alloys. However, the BMGMC sample exhibited more serious localized corrosion, confirming its lower corrosion resistance compared with the monolithic BMG.

The surface corrosion products detected in both the monolithic BMG and BMGMC samples mainly contained O, Zr, Cu, Ag, Al, and Cl. Ta was detected only in the BMGMC sample. In both alloys, the contents of Zr and Al on the corroded surface, as shown in Figure 6, and in the near-surface cross-sectional regions, as shown in Figure 7, were significantly reduced. This indicates the preferential dissolution of Zr and Al during Cl^−^-induced corrosion, whereas Cu and Ag remained relatively stable. As mentioned above, elemental dissolution generally proceeds through the formation of metal–chloride complexes [23,24,25,26]. The standard enthalpies of formation at 298.15 K for ZrCl_4_, CuCl_2_, AlCl_3_, and AgCl are −980.5, −220.1, −704.2, and −127.0 kJ/mol, respectively [27]. These values suggest that Zr and Al are more prone to dissolution than Cu and Ag, which explains the depletion of Zr and Al in the corrosion-affected regions.

In addition, Cu-rich crystalline deposits were observed on the inner walls of the pits in both the monolithic BMG and BMGMC samples, as shown in Figure 6a,b. These deposits were likely formed through the re-deposition of dissolved Cu ions [17]. Since Cu is a relatively noble element in these alloys, the formation of Cu-rich deposits may further accelerate the dissolution of the surrounding regions that remain enriched in Zr and Al. This effect can be attributed to the formation of local galvanic cells between the Cu-rich deposits and the adjacent glassy matrix [17]. The EDS composition analysis results in Table 2 also confirm that the Cu-rich wall at points 1 and 3 in Figure 6a,b has a high Cu content and also identified Ta precipitates at point 5 in Figure 6c.

Moreover, more severe corrosion was observed in the BMGMC sample near the Ta precipitates, as shown in Figure 6c. The Ta precipitate/glassy matrix interface is a vulnerable region where Cl^−^ ions can more easily penetrate and break down the passive film. After pit initiation, the exposed glassy matrix serves as a local anodic region, while the adjacent passive Ta precipitates act as cathodic sites. This local potential difference promotes galvanic coupling and accelerates matrix dissolution. Consequently, the corrosion attack is not only driven by Cl^−^ penetration but also enhanced by galvanic corrosion. This combined effect causes the corrosion to propagate preferentially along the glassy matrix surrounding the Ta precipitates, leading to deeper penetration, wider attack regions, and more serious local damage. The EDS results in Table 2 also identified points 6 in Figure 6c as corrosion products following chlorine attack.

Figure 7 and Table 3 present cross-sectional observations and EDS compositional analyses of the small pits and corrosion products formed on the monolithic BMG and BMGMC. As shown in Figure 7a, point 1 corresponds to a corrosion-product region on the monolithic BMG. The EDS results reveal relatively high O and Cu contents, suggesting that Cu migrated into the corroded region and became incorporated into the oxidation products, resulting in the formation of a Cu-rich phase. In contrast, point 2 is located in the unaffected substrate and therefore retains relatively high Zr and Cu contents.

For the BMGMC shown in Figure 7b, point 3 contains 100 wt.% Ta. This Ta-rich region is considered to originate from a Ta particle exposed from within the BMGMC matrix during corrosion. Point 4 also exhibits elevated Cu and oxygen contents, indicating the formation of Cu-rich oxidation products. The Cu content of this Cu-rich region is higher than that observed in the monolithic BMG, suggesting that the oxidation reaction is more pronounced in the BMGMC. Point 5 contains a higher Zr concentration than points 3 and 4 because it is located within the BMGMC matrix.

As shown by the microscopic observations in Figure 6a,b and Figure 7a,b, chloride-induced breakdown of the passive film resulted in pitting corrosion in both the monolithic BMG and the BMGMC exposed to 3.5 wt.% NaCl solution. Previous investigations [28,29] of micron-sized Ta particles embedded in Zr-based bulk metallic glass composites revealed the formation of a metastable ZrCu phase on or near the surfaces of micron-sized Ta particles, with the Ta particles potentially serving as heterogeneous nucleation sites for its precipitation.

**Figure 8:**
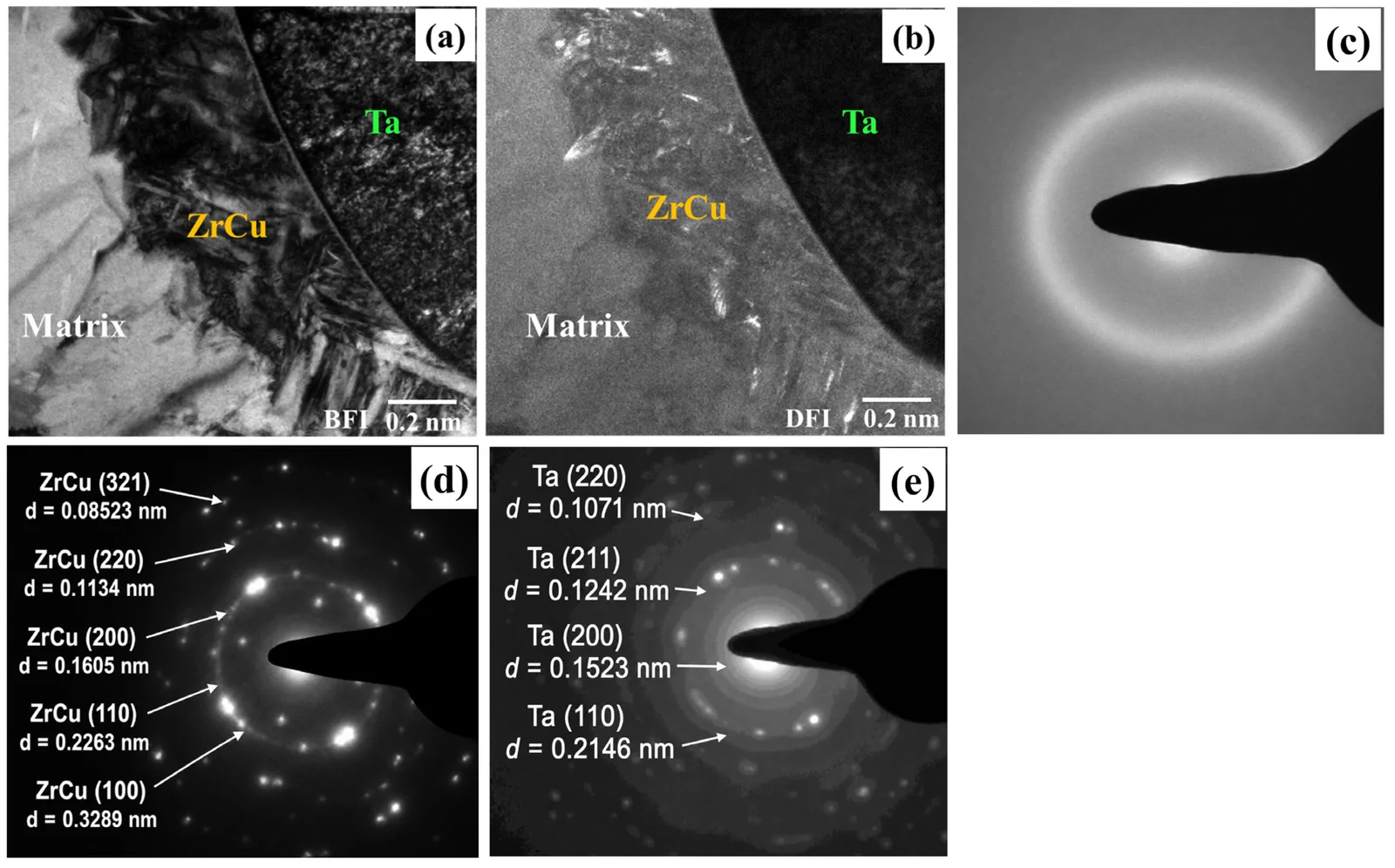
TEM micrographs of the BMGMC after exposure to a 3.5 wt.% NaCl solution (**a**) bright-field image (BFI), (**b**) dark-field image (DFI), (**c**) selected area diffraction (SAD) pattern of the amorphous matrix, (**d**) selected area diffraction (SAD) pattern of the ZrCu phase, and (**e**) selected area diffraction (SAD) pattern of Ta phase.

To establish the initial microstructural features associated with the subsequent corrosion behavior, the Ta particle and its surrounding region were examined by transmission electron microscopy before the corrosion test, and the corresponding observations were obtained from our earlier studies [28,29]. The bright-field and dark-field TEM images in Figure 8a,b, respectively, confirm the presence of needle-like ZrCu precipitates adjacent to the Ta particle. After the corrosion test, a substantial amount of corrosion products was observed around the Ta particles (in Figure 6c). The matrix of the BMGMC alloy was identified as an amorphous matrix, thus exhibiting diffraction rings, as shown in the select area diffraction (SAD) pattern in Figure 8c. Considering the pre-existing ZrCu phase, identified with a SAD pattern in Figure 8d, these corrosion products are inferred to be associated with the preferential dissolution of the ZrCu precipitates surrounding the Ta particles. Figure 8d shows the identification of the SAD pattern in the Ta phase. The needle-like morphology of the ZrCu phase provides a relatively large interfacial area and numerous locally active sites, making it more susceptible to localized corrosion than the Ta phase. Once the ZrCu phase is dissolved, the surrounding amorphous matrix becomes exposed. The electrochemical potential difference between the corrosion-resistant Ta particles and the amorphous matrix then promotes galvanic coupling, with the Ta particles acting as relatively cathodic sites and the matrix undergoing accelerated anodic dissolution. These observations therefore suggest that corrosion initially occurred through preferential dissolution of the ZrCu phase, followed by galvanic corrosion between the Ta particles and the surrounding amorphous matrix.

### 3.4. Corrosion Mechanism of the Monolithic BMG and BMGMC in 3.5 wt.% NaCl Solution

**Figure 9:**
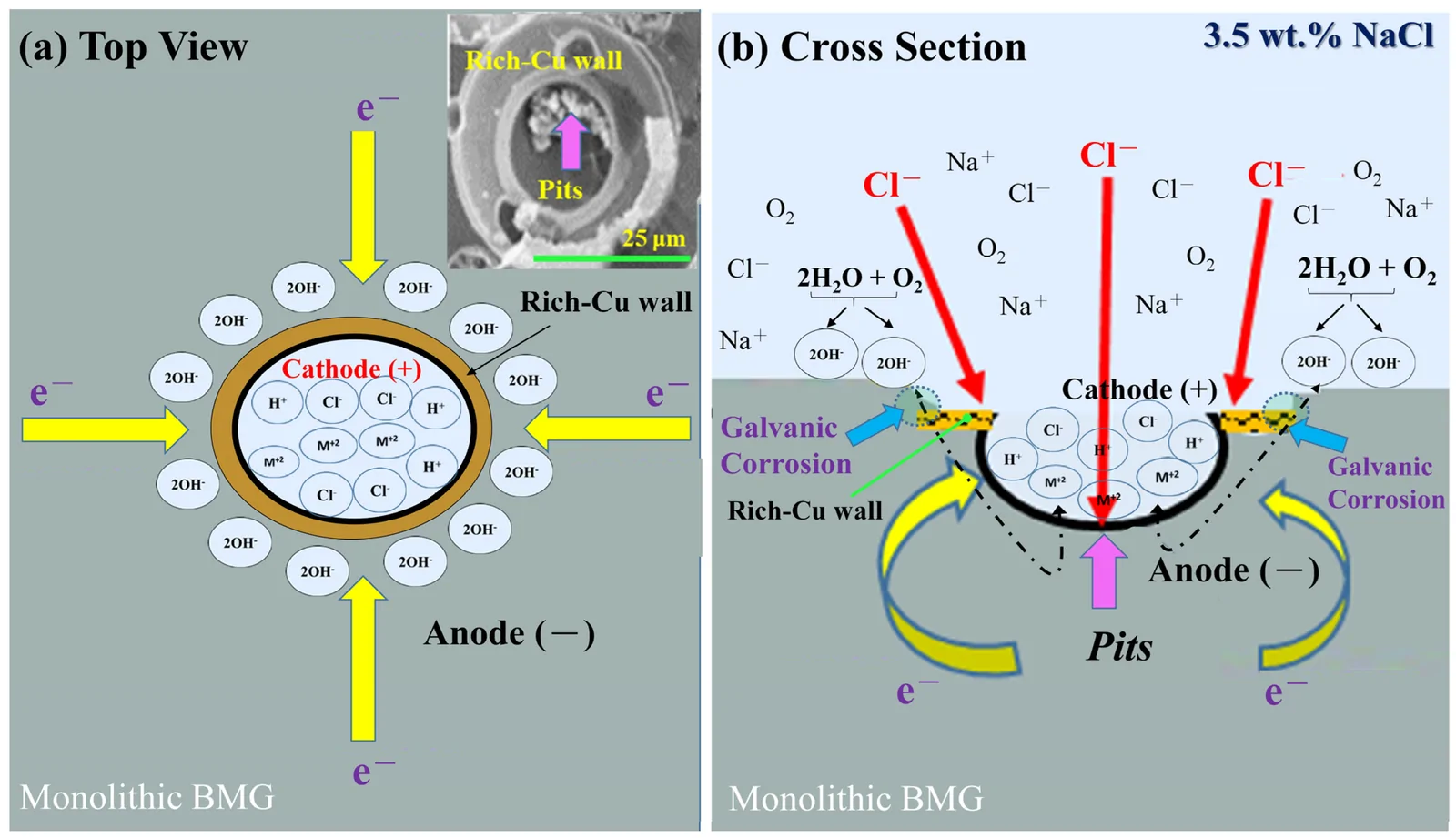
Schematic illustration of the corrosion mechanism of the monolithic BMG in 3.5 wt.% NaCl solution: (**a**) top view and (**b**) cross-sectional view.

Figure 9 schematically summarizes the corrosion mechanism of the monolithic BMG in 3.5 wt.% NaCl solution. During the initial stage of corrosion, the surface of the monolithic BMG is protected by a passive film mainly associated with Zr-based oxides. The formation of ZrO_2_ through anodic oxidation can be represented as:

Zr + 2H_2_O → ZrO_2_ + 4H^+^ + 4e^−^ (1)

With continued exposure to the chloride-containing solution, Cl^−^ ions progressively attack local weak sites in the passive film and promote its breakdown. Once the protective film is locally damaged, anodic dissolution of the exposed metallic constituents occurs. This process can be generally expressed as:

*M* → *M*^n+^ + *n*e^−^ (2)

where M represents a metallic element in the BMG matrix. Because Zr is the major constituent of the alloy, its anodic dissolution can be specifically represented as:

Zr → Zr^4+^ + 4e^−^ (3)

At the same time, the electrons generated by anodic dissolution are consumed by the cathodic oxygen-reduction reaction in the naturally aerated NaCl solution:

O_2_ + 2H_2_O + 4e^−^ → 4OH^−^ (4)

The simultaneous anodic and cathodic reactions sustain the electrochemical corrosion process. As the passive film continues to deteriorate, small corrosion pits form and subsequently grow on the specimen surface. During pit development, preferential dissolution of the more electrochemically active constituents, particularly Zr, results in relative Cu enrichment in and around the corroded regions. This behavior is consistent with the Cu-rich regions observed around the corrosion pits. As corrosion progresses, the compositional difference between the Cu-enriched region and the surrounding BMG matrix increases the local electrochemical heterogeneity, which may promote galvanic interactions and further accelerate localized corrosion.

**Figure 10:**
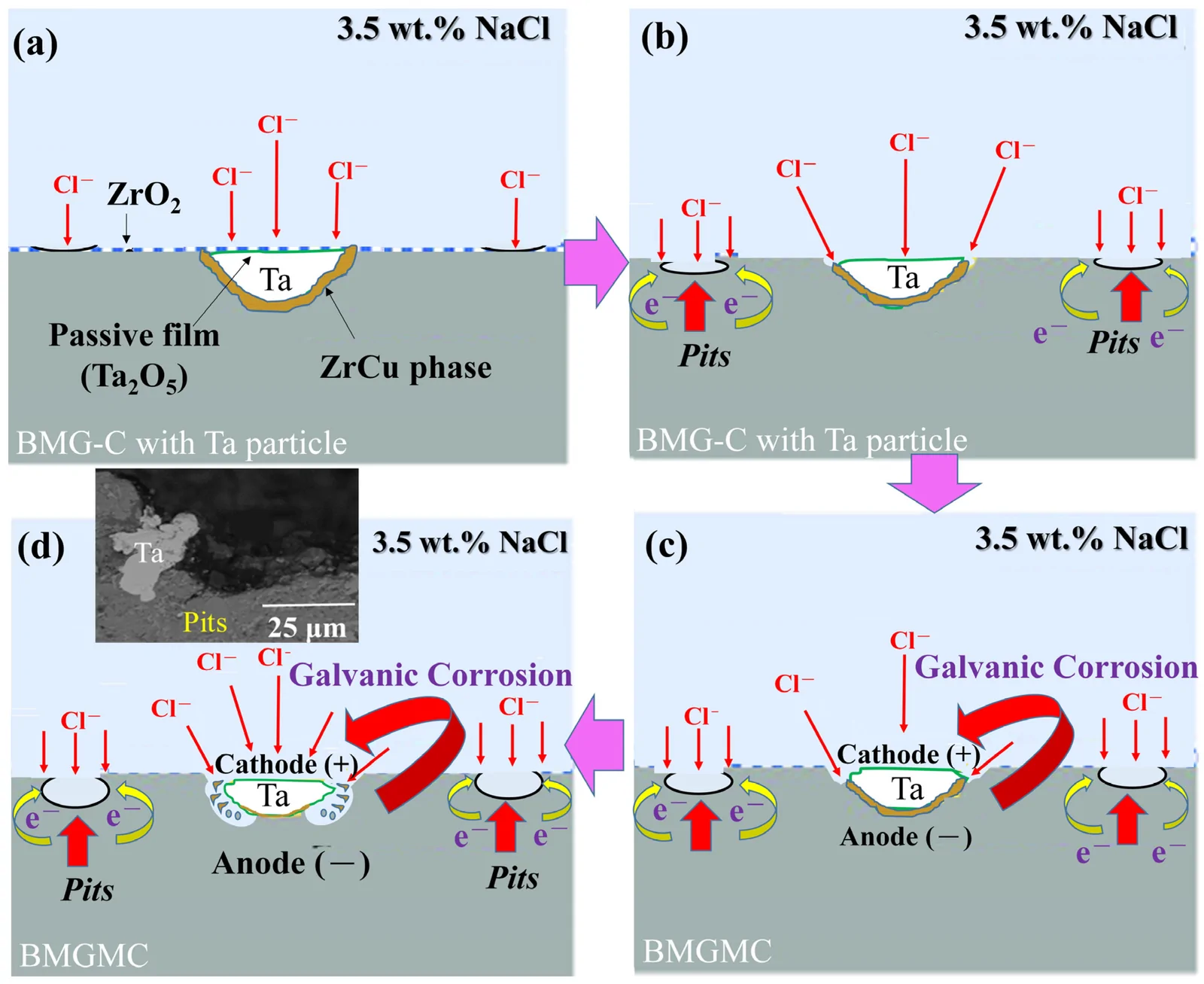
Schematic illustration of the corrosion mechanism of the BMGMC in 3.5 wt.% NaCl solution: (**a**) initial stage, (**b**) passive film breakdown, (**c**) occurrence of galvanic corrosion, and (**d**) final corrosion stage.

Figure 10 shows the proposed corrosion mechanism of the BMGMC in 3.5 wt.% NaCl solution. Similar to the monolithic BMG, corrosion begins with chloride-induced local breakdown of the Zr-rich passive film on the BMGMC surface, as illustrated in Figure 10a. The anodic dissolution and cathodic oxygen-reduction reactions can likewise be described by Equations (2)–(4). Following passive-film breakdown, a relatively large pit develops in the more severely attacked region, while numerous smaller pits form over the surrounding surface.

At the same time, the Ta-rich regions exhibit comparatively high electrochemical stability because Ta is readily passivated by a stable Ta_2_O_5_-based oxide film. The anodic formation of Ta_2_O_5_ can be represented schematically as:

2Ta + 5H_2_O → Ta_2_O_5_ + 10H^+^+ 10e^−^ (5)

Consequently, the Ta-rich regions remain relatively resistant during the initial stage of chloride attack, as illustrated in Figure 10b.

With further corrosion, the ZrCu phase becomes exposed at the corroded surface. Preferential dissolution of Zr from the ZrCu-containing region may be represented schematically by Equation (3), resulting in relative enrichment of Cu in the remaining corroded region. Previous observations of Cu enrichment in Zr-based BMGs exposed to chloride-containing solutions are consistent with such selective dissolution behavior. Because of the electrochemical difference between the ZrCu phase and the adjacent amorphous matrix, local galvanic coupling develops and promotes preferential dissolution around these heterogeneous regions, as shown in Figure 10c.

As chloride attack continues, localized corrosion becomes increasingly severe. In the later stage, extensive dissolution of the ZrCu-containing region leads to the formation of fractured corrosion products and debris around the main pit, as illustrated in Figure 10d. At the same time, additional small pits continue to develop in the surrounding area. Thus, although the Ta-rich phase exhibits relatively high resistance during the initial stage, the heterogeneous microstructure consisting of the Ta phase, ZrCu phase, and amorphous matrix produces local electrochemical differences that contribute to the development and propagation of localized corrosion in the BMGMC.

Although the above results provide insight into the corrosion mechanism of the BMG and BMGMC, several limitations of the present study should be acknowledged. The corrosion behavior was evaluated mainly in 3.5 wt.% NaCl solution at room temperature; therefore, the influence of other environmental factors, such as chloride concentration, pH, and temperature, was not considered. In addition, although the TEM and electrochemical results provide evidence for the roles of the Ta and ZrCu phases in localized corrosion, the chemical composition and evolution of the passive films were not directly characterized by surface-sensitive techniques such as XPS. The local electrochemical potential differences among the Ta phase, ZrCu phase, and amorphous matrix were also not directly measured. Further studies combining surface chemical analysis and local electrochemical measurements would provide a more complete understanding of the corrosion mechanism of the BMG composite.

## 4. Conclusions

In this study, the corrosion behavior of a monolithic (Zr_48_Cu_36_Al_8_Ag_8_)Si_0.75_ BMG and a (Zr_44_Cu_36_Al_8_Ag_8_Ta_4_)Si_0.75_ BMGMC were examined in 3.5 wt.% NaCl solution. XRD, SEM, and TEM were first used to clarify the as-cast microstructures, and the corrosion response was then evaluated by potentiodynamic polarization, surface observation, and post-corrosion SEM–EDS analysis. The main conclusions are summarized as follows:

1. The as-cast Zr_48_Cu_36_Al_8_Ag_8_Si_0.75_ alloy exhibited a predominantly amorphous structure, as evidenced by the broad diffuse halo in the XRD pattern and the absence of discernible crystalline features in the SEM images.
2. The addition of Ta resulted in the formation of a BMGMC with Ta particles embedded in a predominantly amorphous matrix.
3. In 3.5 wt.% NaCl solution, both the monolithic BMG and the BMGMC exhibited passivation behavior during potentiodynamic polarization testing, indicating the formation of a protective surface film.
4. The BMGMC exhibited a lower corrosion current density than the monolithic BMG, indicating a lower corrosion rate at the initial stage of the electrochemical reaction.
5. Although the BMGMC exhibited a lower initial corrosion rate, its narrower passive region and lower pitting resistance indicated a greater susceptibility to localized corrosion at higher applied potentials.
6. The selected-area diffraction (SAD) pattern confirmed the presence of the ZrCu phase, Ta phase, and amorphous matrix. TEM bright-field and dark-field images further revealed needle-like ZrCu precipitates adjacent to the Ta particles.
7. In the BMGMC, chloride attack preferentially dissolved the ZrCu-containing regions near the Ta/ZrCu/amorphous-matrix interfaces. After breakdown of the passive film, galvanic coupling between the Ta particles and the amorphous matrix further enhanced local dissolution and promoted the development of more severe pitting corrosion.

## Figures and Tables

**Table 1:** Electrochemical corrosion parameters of the monolithic BMG and BMGMC derived from the polarization data shown in Figure 2.

Materials	Corrosion Index			
	E_corr_ (V vs. SCE)	i_corr_ (A/cm^2^)	E_pit_ (V vs. SCE)η	η_pit_ = E_pit_ − E_corr_ (V)
Monolithic BMG	−0.401	2.8 × 10^−6^	0.794	1.195
BMGMC	−0.495	1.2 × 10^−7^	0.495	0.990

Note: E_corr_, i_corr_, E_pit_, and η_pit_ represent the corrosion potential, corrosion current density, pitting potential, and pitting resistance interval, respectively. A more positive E_corr_, lower i_corr_, higher E_pit_, and larger η_pit_ indicate better corrosion resistance.

**Table 2:** EDS analysis results of the corroded surface obtained from the regions shown in Figure 6.

Regions			Element (at.%)					
		O	Al	Cl	Cu	Zr	Ag	Ta
**Monolithic BMG**	**1**	28.3	0	0	62.5	4.5	4.7	0
	**2**	23.3	3.5	0	42.1	26.9	4.2	0
**BMGMC**	**3**	18.2	0	0	75.2	4.6	2.0	0
	**4**	16.0	4.2	0	46.7	28.2	4.9	0
	**5**	0	0	0	0	0	0	100
	**6**	44.0	0.8	0.4	46.4	6.5	1.6	0.2

**Table 3:** EDS analysis results of the corroded surface obtained from the regions shown in Figure 7.

Regions		Element (at.%)					
		O	Al	Cu	Zr	Ag	Ta
**Monolithic BMG**	**1**	30.5	2.5	43.1	19.3	4.6	0
	**2**	0	7.4	36.2	48.0	8.4	0
**BMGMC**	**3**	0	0	0	0	0	100
	**4**	47.5	2.7	33.7	10.5	5.6	0
	**5**	0	7.5	36.3	44.0	8.2	4.0

## Data Availability

The original contributions presented in this study are included in the article. Further inquiries can be directed to the corresponding authors.
